# Epidemiology of *Staphylococcus aureus* Non-Susceptible to Vancomycin in South Asia

**DOI:** 10.3390/antibiotics12060972

**Published:** 2023-05-27

**Authors:** Mohammad Ejaz, Muhammad Ali Syed, Charlene R. Jackson, Mehmoona Sharif, Rani Faryal

**Affiliations:** 1 Department of Microbiology, Government Postgraduate College Mandian Abbottabad, Abbottabad 22044, Pakistan; m.ejaz@bs.qau.edu.pk; 2 Department of Microbiology, Qauid-i-Azam University, Islamabad 45320, Pakistan; mehmoona@bs.qau.edu.pk (M.S.); ranifaryal@qau.edu.pk (R.F.); 3 Department of Microbiology, The University of Haripur, Haripur 22620, Pakistan; syedali@uoh.edu.pk; 4 U.S. Department of Agriculture, Agricultural Research Service, U.S. National Poultry Research Center, Poultry Microbiological Safety and Processing Unit, Athens, GA 30605, USA

**Keywords:** *Staphylococcus aureus*, antibiotic resistance, vancomycin, vancomycin-intermediate resistant *S. aureus*, vancomycin-resistant *S. aureus*

## Abstract

*Staphylococcus aureus* is one of the ESKAPE (*Enterococcus faecium*, *Staphylococcus aureus*, *Klebsiella pneumoniae*, *Acinetobacter baumannii*, *Pseudomonas aeruginosa*, and *Enterobacter* species) pathogens among which multidrug resistance has emerged. Resistance to methicillin has resulted in clinicians using the antibiotic of last resort, vancomycin, to treat infections caused by methicillin-resistant *S. aureus* (MRSA). However, excessive use and misuse of vancomycin are major causes of resistance among *S. aureus* strains. South Asia encompasses ~25% of the world’s population, and countries in South Asia are often characterized as low- and middle-income with poor healthcare infrastructure that may contribute to the emergence of antibiotic resistance. Here, we briefly highlight the mechanism of vancomycin resistance, its emergence in *S. aureus*, and the molecular epidemiology of non-susceptible *S. aureus* to vancomycin in the South Asian region.

## 1. Introduction 

*Staphylococcus aureus* exists as a commensal of normal human microbiota, colonizing approximately 20–40% of healthy individuals [1]. However, *S. aureus* is also a widespread human pathogen that causes a range of community-acquired and nosocomial infections, ranging from minor skin and soft tissue infections to more serious and deadly conditions such as pneumonia, bacterial endocarditis, osteomyelitis, toxic mediated syndromes, and bacteremia [2,3,4]. Due to its metabolic flexibility, *S. aureus* adapts well to different environmental conditions and thus remains a major public health concern. With the advent of antibiotics such as penicillin and methicillin in the 20th century, *S. aureus* infections were effectively treated. However, resistance to both of these drugs emerged rapidly, resulting in difficulty in the treatment of infections [5]. During the last century, various reports have indicated a significant increase in healthcare-associated costs due to the emergence of antibiotic resistance in *S. aureus* strains [6]. Both hospital-associated methicillin-resistant *Staphylococcus aureus* (HA-MRSA) and community-associated MRSA (CA-MRSA) are major health concerns, and are on the rise globally [7]. The majority of HA-MRSA strains from various countries share the same genotype, as shown by molecular epidemiology research [8,9]. This suggests that a few healthcare-associated clones from Asia have spread internationally. However, a few relatively high-income countries in Asia, such as Taiwan, Japan, Korea, Hong Kong, and Singapore, have produced the majority of studies [9,10,11]. 

South Asia is the southern region of the continent Asia and consists of eight countries, including Pakistan, India, Bangladesh, Sri Lanka, Nepal, Maldives, Afghanistan, and Bhutan. South Asia covers about 2 million square miles, representing 3.5% of the world’s land area and 11.71% of the continent of Asia [12]. This region of South Asia has the highest population density on the entire planet, contributing to 24.89% of the world population, i.e., about 1.9 billion individuals [13]. The healthcare system of South Asia is amongst the world’s worst healthcare systems due to poor economic inequalities, and these inequalities compound the widespread gaps in access to healthcare systems [14]. Limited access to healthcare facilities and indiscriminate misuse of antibiotics are common practices in the region, contributing to the high burden of antimicrobial resistance to various antibiotics. 

Vancomycin became the drug of choice for the effective treatment of MRSA in the late 1980s and is used to treat MRSA infections worldwide. However, *S. aureus* acquired resistance against vancomycin and became a great concern to global public health [15]. Several studies from South Asian countries, including India, Pakistan, Bangladesh, Sri Lanka, and Nepal, reported either reduced susceptibility or complete resistance to vancomycin, while no studies have been published from Afghanistan, Maldives, or Bhutan on the emergence of non-susceptible *S. aureus* strains against vancomycin [16,17,18,19]. 

Our understanding of the epidemiology of staphylococcal infections has been significantly constrained by the information being either incomplete or completely unavailable for the resource-constrained countries of Southeast Asia and South Asia. A recent systematic review reported a prevalence of 1.2% vancomycin-resistant *S. aureus* (VRSA) in Asia, 1.1% in Europe, 3.6% in America, and 2.5% in Africa [20]. Therefore, this review highlights the epidemiology of vancomycin-intermediate resistant *S. aureus* (VISA), VRSA, and heterogeneous vancomycin-intermediate resistant *S. aureus* (hVISA) in the South Asian region. 

## 2. Antibiotic Resistance in *S. aureus*

Indiscriminate use of antibiotics played a significant role in the emergence of resistant bacteria, the increase in multi-drug resistant strains, and the spread of resistance among bacterial species [21]. Resistance to antibiotics in *S. aureus* is attributed to chromosomal mutations and extra-chromosomal elements such as plasmids. Mobile genetic elements such as staphylococcal cassette chromosomes, plasmids, bacteriophages, transposons, and pathogenicity islands are involved in horizontal gene transfer, which plays a significant role in the development and dissemination of antibiotic resistance in bacteria [22].

Several waves represent the emergence of antibiotic resistance in *S. aureus* including the emergence of penicillin-resistant *S. aureus* (PSRA) followed by MRSA, CA-MRSA, HA-MRSA, VISA, and VRSA, respectively [23]. PSRA was reported after a few years of treatment in the early 1940s, and its infections in humans spread across both community and hospital settings in the 1950s to early 1960s [5,24]. The *blaZ* gene, which encodes a β-lactamase, primarily conferred resistance in *S. aureus* by hydrolyzing the β-lactam ring of the antibiotic, thus inactivating penicillin [25]. After the emergence of PRSA, methicillin was introduced to treat the infections. Despite its efficacy in treating the PRSA infection, methicillin resistance soon emerged, and MRSA strains were reported consistently within two years of its use in clinical practice [26]. Currently, MRSA has spread worldwide, accounting for a large number of CA-MRSA and HA-MRSA infections and causing increased morbidity and mortality. Several surveys in different countries have reported the number of deaths caused by MRSA [27,28,29]. In America alone, ~19,000 hospitalized patients have died per year due to MRSA, while over 17,000 patients infected with *S. aureus* died every year due to antimicrobial resistance (AMR) against β-lactam antibiotics [30,31]. The emergence of CA-MRSA outside of healthcare settings in healthy individuals has led to complications in managing infections [32]. All MRSA strains have staphylococcal cassette chromosome (SCC), which carries the *mec*A gene. This gene encodes low-affinity penicillin-binding protein 2a, imparting resistance to β-lactam antibiotics [33]. Staphylococcal cassette chromosome *mec* (SCC*mec*) is the mobile genetic element of *S. aureus*, playing a significant role in staphylococci pathogenesis. SCC*mec* and *mec*A genes are incorporated into the chromosome of *S. aureus,* causing the evolution of resistance and resulting in the emergence of MRSA [34]. The two genes, namely, the cassette chromosome recombinase *(ccr)* gene and the *mec* gene complex, combine to form the SCC*mec* gene [35]. In addition, a new type of *mec* gene, *mec*C, with 63% residue identity to *mec*A, was discovered and found to be predominantly associated with a single lineage of MRSA in European countries [36]. About 615 MRSA strains carry this SCC*mec* gene and have been isolated in 11 different countries of Asia [37]. Eleven different SCC*mec* types have been reported. Of those, SCC*mec* types IV and V are typically carried by CA-MRSA, while SCC*mec* types I, II, and III typically belong to HA-MRSA [33,38,39]. Moreover, CA-MRSA strains carry genes such as *luk*S-PV and *luk*F-PV, which encode a bi-component pore-forming cytolytic toxin, Panton–Valentine leukocidin (PVL), that targets neutrophils, monocytes, and macrophages [38,40].

Since the 1980s, the preferred medication for the treatment of severe infections caused by MRSA has been vancomycin [41]. In 1997, the reduced susceptibility against vancomycin (VISA, minimum inhibitory concentration (MIC) = 8 µg/mL) was reported in Japan [42]. Shortly after the VISA report in Japan, two cases were reported in America, while the first VRSA case emerged in 2002 and was reported in a diabetic patient [43,44]. Since then, several reduced susceptible and resistant strains of *S. aureus* have been reported worldwide. Two methods for molecular characterization of *S. aureus* are widely used, including multilocus sequence typing (MLST) and *spa* typing. MLST is a molecular typing technique used to identify short internal fragments of nucleotide sequences (usually 400–500 bp) in multiple housekeeping genes, while *spa* typing is a rapid and affordable technique with highly discriminatory abilities to determine the variability in the polymorphic X region of staphylococcal protein A gene (*spa* gene), found in all strains of *S. aureus* [45,46]. Non-susceptible vancomycin *S. aureus* isolates were tested by Shekarabi et al., who found three different sequence types (ST) (ST5, ST8, and ST239) to be associated with VRSA [47]. *Spa* typing revealed that *spa* types t030 and t70 belonged to VISA, while t008, t37, and t002 were present in VRSA strains. Shekarabi et al. also showed the MIC values for specific types of VRSA strains and found that ST239-SCC*mec* III/t030 and ST22-SCC*mec* IV/t790 strains had MIC values ≥ 8 μg/mL, ST8-SCC*mec*IV/t008 strains had MIC values of 64 μg/mL, and ST239-SCC*mec* III/t037 and ST5-SCC*mec* II/t002 strains had MIC values of 512 μg/mL [47]. ST239 is endemic in the Asian region and is divided into three different clades: the European, Asian, and South American clades. Moreover, multi-resistant ST239 is more prevalent in Asia, the USA, and European countries [48]. Several studies have reported ST239 MRSA strains that are non-susceptible to vancomycin (VISA and VRSA) in this region [49,50].

### 2.1. Vancomycin’s Mechanism of Action against S. aureus and the Emergence of Resistance

Vancomycin exerts bactericidal activity by interrupting the cell wall synthesis of the bacteria, but many bacteria have overcome its effect and become resistant due to multiple mutations in chromosomal genes that affect cell wall synthesis [51]. The hydrophilic molecules of vancomycin interact with the terminal D-alanyl-D-alanine (d-Ala-d-Ala) moieties of the precursor lipid II through hydrogen bonding. Vancomycin binding causes a conformational change that prevents the inclusion of the precursor in the developing peptidoglycan chain and the subsequent trans-peptidation, resulting in cell wall lysis [15]. 

**Figure 1:**
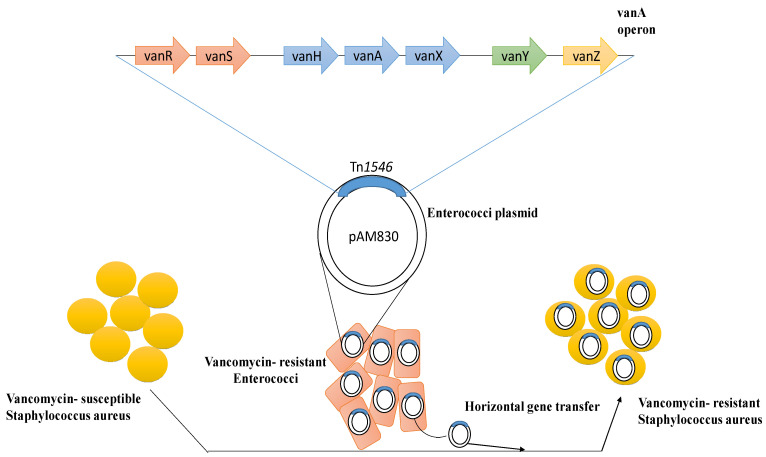
Mechanism of emergence of vancomycin resistance among susceptible *Staphylococcus aureus* through horizontal gene transfer.

However, the increased usage of vancomycin has resulted in the establishment of two other forms of *S. aureus* that show reduced susceptibility or resistance to glycopeptides. The first one, known as VISA, has a thicker cell wall, along with insufficient cross-linking in the cell wall, which causes a buildup of acyl-d-alanyl-d-alanine (X-d-Ala-d-Ala) targets that entrap glycopeptides at the periphery. The second form is VRSA, which is due to transposon Tn1546-carrying the *van*A operon acquired from *Enterococcus* species, resulting in the establishment of high-level resistance (Figure 1) [52,53,54]. The risk of transmission to *S. aureus* and other susceptible medically significant microbes due to the vancomycin-resistant enterococci is high [15].

The first hVISA strain to be identified was Mu3, which was found in a patient with pneumonia in Japan in 1996 who had not responded to vancomycin [28]. The first instance of VISA (Mu50) was documented in a patient who had undergone heart surgery for a surgical wound infection in 1997 [42]. Vancomycin-intermediate resistant strains exhibit certain characteristics, such as reduced autolysis, weakened virulence, and thickened cell walls. The potential resistance is due to an increase in cell wall turnover, which generates non-cross-linked d-anyl-d-alanine side chains, resulting in less exposure of vancomycin to intracellular target molecules [55]. The reported VISA strains evolved from HA-MRSA are mainly from clonal complex 5 or 8 (CC5/CC8), in particular, from ST239 and ST5. However, the MSSA-derived CA-MRSA clone USA300-derived VISA was also reported [56,57]. With the high incidence of MRSA and increased reliability of the use of glycopeptides in the South Asian region, the emergence of non-susceptible strains of *S. aureus* was not surprising. However, non-susceptible strains have not disseminated widely in south Asia, but have been detected sporadically in patients who have previously used vancomycin or were on long-term vancomycin therapy for persistent *S. aureus* infections [58].

### 2.2. Interpretation Criteria for Vancomycin-Susceptible S. aureus

Vancomycin susceptibility testing can be conducted using different methods, including the E test, broth micro-dilution, agar dilution method, etc. The Clinical Laboratory Standards Institute (CLSI) decreased the clinical susceptible MIC breakpoint from 4 μg/mL to 2 μg/mL in 2006 [59]. VRSA has a MIC of >16 µg/mL; VISA a MIC range of 4–8 µg/mL; and hVISA a MIC range of ≤2 µg/mL, with a subpopulation of MIC = 4–8 µg/mL, which could all be classified as *S. aureus* with reduced susceptibility towards vancomycin [60]. The MIC to vancomycin of clinical *S. aureus* isolates has continuously increased with increasing vancomycin prescription, which may lead to more vancomycin treatment failures [59,61].

Due to the presence of hVISA and the inadequacy of standard clinical antimicrobial susceptibility testing to precisely identify VISA, there were still troubling issues even after these interpretation breakpoints were lowered to assure concordance with clinical treatment feedback. Since 2009, it has been impossible to evaluate the vancomycin susceptibility of *S. aureus* using the disc diffusion method, since it is unable to distinguish between vancomycin-susceptible *S. aureus* (VSSA), VISA, and VRSA strains [62]. Clinical laboratories in Taiwan use automated platforms such as the BD PhoenixTM automated testing system, the VITEK^®^ 2 automated instrument, and the MicroScan system because the time-consuming and labor-intensive standard dilution methods (micro- or macro-dilution and agar dilution) are not appropriate for routine mass clinical use. One of the techniques suggested by CLSI to identify vancomycin resistance in *S. aureus* is vancomycin screening agar, which includes vancomycin at 6 µg/mL and is being widely used in the region to determine vancomycin-resistant enterococci (VRE). However, there is the possibility that this screening may overlook VISA strains with vancomycin MICs of 4–6 μg/mL [63].

### 2.3. Selection Criteria

A comprehensive literature search was performed on the PubMed, Google Scholar, and Web of Science databases for original research publications from 1997 to March 2023. The terms used in the search strategy included vancomycin-resistant *Staphylococcus aureus*, vancomycin intermediate *Staphylococcus aureus*, heterogeneous vancomycin *Staphylococcus aureus*, VRSA, VISA, and hVISA in each of the South Asian countries. The bibliographies of relevant articles were also searched to identify other related studies. The number of publications found to be relevant to the epidemiology of non-susceptible vancomycin *S. aureus* (VISA, VRSA, hVISA) in South Asia was <40, showing the lack of reporting in the region, especially in India and Pakistan, where the AMR burden is alarmingly high. 

## 3. Vancomycin Non-Susceptible *S. aureus* in South Asia

### 3.1. Pakistan 

Since the emergence of VRSA in the USA, many other countries have also reported vancomycin resistance in clinical isolates of MRSA. Azhar et al. reported 7.3% VRSA in the clinical isolates, and the *van*A operon was detected in 74% of those VRSA isolates [64]. A study reported the persistence of VRSA in the blood of postoperative cardiac surgery patients, and isolates were found to carry *van*A on a plasmid and *ica*A on the chromosome [65]. Under conditions of sub-inhibitory vancomycin concentrations, the isolated strain also formed some extracellular polymeric matrix substance and showed resistance to triton-X100-caused autolysis [65]. A study conducted by Taj et al. in Pakistan identified one strain as VRSA (MIC = 32 µg/mL) and four strains as VISA (two strains with MIC = 8 µg/mL and two strains with MIC = 16 µg/mL) [66]. A retrospective study conducted in Pakistan reported a prevalence of 8.33% VRSA (*n* = 565) and 11.68% VISA (*n* = 792), which was the highest reported frequency found so far in Pakistan [67]. A prevalence of 13% VISA in the clinical isolates of MRSA strains was reported in a study conducted in Karachi, Pakistan [68]. In 2019, Saeed et al. [69] selected 30 isolates that were phenotypically resistant to methicillin and vancomycin to find resistance genes. Out of 30 MRSA isolates, 10 (33.33%) were successfully amplified for the *mec*A partial region of the antibiotic resistance gene, while *van*A was amplified from 14 (46.6%) isolates. Similarly, another study was conducted in which a total of 86 VRSA isolates were subjected to the amplification of vancomycin-resistance encoding genes. It was found that 40 isolates showed the presence of *van*A, whereas 51 isolates showed the presence of *van*B. Most of the isolates that were *van*B-positive also contained *van*A [16]. The resistant strains of *S. aureus* against methicillin and vancomycin were reported in a study conducted in a tertiary care hospital of Lahore, Pakistan. The prevalences of MRSA (25.5%), VISA (11%), and VRSA (2.5%) were reported in patients with skin injuries [70]. A similar study evaluated the antimicrobial resistance patterns of *S. aureus* isolated from different clinical samples and found a presence of 12% VRSA [71]. Table 1 presents the epidemiology *S. aureus* strains that exhibit non-susceptibility to vancomycin in South Asian countries, including Pakistan.

### 3.2. India

A routine nasal carriage survey in the intensive care unit of a hospital in northern India identified two VISA isolates (MIC = 8 mg/L) carrying the *van*A gene [72]. The study was considered as the first study from India to report the presence of the *van*A gene among VISA isolates. The asymptomatic colonization of *van*A-positive VRSA raised apprehension about the spread of resistant strains among hospital staff and the local community. The study by Goud et al. in India reported a 1.4% VRSA prevalence confirmed by the presence of *van*A in the swab samples collected from the anterior nares, forearms, and hands of 1000 healthy individuals [73]. Vancomycin resistance in MRSA isolates from the ICUs of tertiary care hospitals in Hyderabad, India was tested, and the presence of 16 VISA (MIC = 4–8 mg/L) and 7 VRSA (MIC = 16–64 mg/L) strains among the clinical isolates were reported; 6 of these VRSA strains were *van*A-positive. A recent study from India reported the presence of 7 (6.08%) VRSA strains and 53 (46.08%) VISA strains among clinical isolates [74]. A reduced susceptibility of 11.6% (*n* = 33) of the isolates to vancomycin was also observed in Eastern India. Among the 33 strains with reduced susceptibility, 18 were VISA and 13 were hVISA [75]. In 2004, the Asian Network for Surveillance of Resistant Pathogens (ANSORP) reported the presence of hVISA among 1357 MRSA isolates in Asian countries, which showed a prevalence of 2.1% in Thailand, Singapore (2.3%), Vietnam (2.4%), the Philippines (3.6%), South Korea (6.1%), India (6.3%) and Japan (8.2%) [76]. The *spa* type t601, which belongs to CC5, showed a frequency of 33.3% for hVISA, and t002 of CC5 accounted for a 6.9% prevalence among hVISA strains in a study conducted by ANSORP (Asian Network for Surveillance of Resistant Pathogens) in 2004–2006 [77]. A study in Puducherry, India determined the prevalence of 12.4% hVISA among clinical isolates. Moreover, the study also determined the distribution of SCC*mec* types among hVISA isolates and found SCC*mec* type III among 8%, SCC*mec* type IV in 17.7%, and SCC*mec* type V in 50% of the strains [78]. A similar study carried out in India found a prevalence of 7% (*n* = 33) hVISA/VISA strains among the clinical isolates of MRSA and MSSA. Out of these 33 hVISA/VISA strains, 42.4% (*n* = 14) belonged to agr I, 24.2% (*n* = 8) belonged to agr II, 30% (*n* = 10) belonged to agr III, and 3% (*n* = 1) belonged to agr IV [79]. A study from a tertiary care hospital in Coastal Karnataka, India, reported a prevalence of 6.4% hVISA among MRSA isolates [80]. 

### 3.3. Bangladesh

The study conducted in Bangladesh by Islam et al. reported the presence of 2 (13.3%) VRSA strains among the samples collected from wounds of hospitalized patients, and found the presence of *van*B in the isolated strains [81]. Bangladesh also reported the emergence of VRSA among MRSA clinical isolates and detected the presence of vancomycin resistance in 3 (7.89%) of the MRSA isolates. A similar study reported a prevalence of 28% (*n* = 8) VRSA, with 6 strains having MIC values of 32 μg/mL and 2 strains having MIC values of 64 μg/mL, and 55% (*n* = 16) were VISA [19]. The study conducted in 2010–2011 in Bangladesh reported a prevalence of 93.44% VISA among clinical isolates, indicating the growing antimicrobial-resistant strains in the region [82].

*S. aureus* strains isolated from patients in the North Central region of Bangladesh were analyzed for their molecular features and genetic backgrounds. In a study of 430 *S. aureus* clinical isolates, 31% were MRSA, with 73% having SCC*mec* type IV and 14% having type V, and most belonging to coagulase (*coa*) genotypes IIa, IIIa, IVb, and XIa. The most common *coa* type in MSSA was IIIa, followed by Va, IIa, and VIa. The study also reported that CC88 (ST88, ST2884) and ST772 were the potential prevailing lineages of PVL-positive MRSA/MSSA. Additionally, the newly discovered CC80 clade was among the primary PVL-negative MRSA lineages distributed in an endemic manner throughout Bangladesh [83]. A research investigation was conducted to ascertain the prevalence of MRSA and VRSA nasal colonization among healthcare providers. The findings of the study revealed that there was a complete absence of resistance to vancomycin, whereas the prevalence of MRSA was 7.2% [84]. A cross-sectional observational study was conducted at the Department of Microbiology, Mymensingh Medical College, Mymensingh, Bangladesh, to identify the presence of MRSA and its susceptibility to various antibiotics. The research revealed a prevalence rate of 26.4% among clinical isolates, and the isolates exhibited a sensitivity rate of 100% towards vancomycin and gentamicin [85]. In a related investigation carried out by Pervaz et al., it was discovered that the frequency of MRSA was 58.4% (*n* = 38) among clinical samples collected from Dhaka City in Bangladesh. This included 22 cases of CA-MRSA and 16 cases of HA-MRSA. However, all MRSA isolates were observed to be receptive to vancomycin [86].

### 3.4. Sri Lanka

According to a 2004–2006 ANSOPR study, the prevalence of nosocomial *S. aureus* isolates in Sri Lanka was 86.5% [87]. According to a study conducted in 2017 by Jayaweera et al., MRSA had become a severe hazard to Sri Lanka’s public health, and its isolation rate was high (40.2%). All of the MRSA isolates had MICs of less than 2 μg/mL [88]. Out of 100 isolates, 21 (21%) were CA-MRSA and 79 (79%) were HA-MRSA, according to a report from the National Hospital of Sri Lanka [89]. Among the HA *S. aureus* infections, the rates of MRSA were quite high in Sri Lanka (86.5%), while among CA *S. aureus* infections, the proportion of MRSA was 38.8% in Sri Lanka [87]. In 2019, an observational study revealed the presence of PVL-positive ST5-MRSA-IVc belonging to CC5 isolates from Sri Lanka, which is one of the major clones spread in different countries such as Australia, the United Kingdom, and Argentina, and might be a threat to neighboring countries [90]. The first case of reduced susceptibility against vancomycin emerged in 2002 in a 60-year-old man with a surgical site infection [91]. None of the MRSA isolates in any other study reported the emergence of resistance against vancomycin. 

### 3.5. Nepal

A cross-sectional study undertaken for the antimicrobial susceptibility profiling of the isolates from chronic dacryocystitis in 2010 in Nepal showed the emergence of vancomycin resistance in 81.48% (*n* = 22) of the isolated strains of *S. aureus* [92]. Other similar studies were conducted in Nepal, but no other studies reported the emergence of complete valid resistance to vancomycin in *S. aureus* strains. An increase in the MIC values of vancomycin among MRSA strains also emerged in Nepal, and 4 (9.52%) strains of MRSA were reported as VISA in a study conducted in Nepal [93]. 

The prevalence of MRSA in Nepal has shown an upward trajectory, ranging from 29.1% to 68%, as reported in previous studies [94,95,96]. However, there have been few research studies conducted on the identification of VISA, VRSA, and their associated genetic markers. The prevalence of VRSA among MRSA isolates was reported to be 11.11% (*n* = 5) in a hospital-based cross-sectional study conducted at the Annapurna Neurological Institute and Allied Sciences, Kathmandu, Nepal. The VRSA isolates underwent screening to determine the presence of *van*A and *van*B. The results indicated that *van*A was present in two of the isolates, whereas *van*B was not detected in any of the isolates [97]. A research investigation was carried out to ascertain the MICs of vancomycin in 47 strains of MRSA that were obtained from clinical specimens gathered from a medical facility located in Nepal. The results of the study revealed that the MIC values ranged from 0.125 μg/mL to 1 μg/mL, and no VISA or VRSA was detected [96].

### 3.6. Afghanistan

A study was conducted to assess the antimicrobial susceptibility patterns of *S. aureus* strains obtained from patients at two major healthcare centers situated in Kabul, Afghanistan. The study revealed that a significant proportion of the isolates (56.2%) were MRSA, while vancomycin exhibited no resistance against the pathogen [98].

The multidrug-resistant *S. aureus* including MRSA has also been reported in a few studies from war-torn Afghanistan [99,100]. The most commonly detected clones were the Bengal Bay clone (ST772-MRSA-V PVL+), the Southwest Pacific clone (CC30-MRSA-IV PVL+), and the CC22-MRSA-IV TSST-1+ clone [99]. No specific study from Afghanistan has reported the emergence of vancomycin non-susceptible *S. aureus*. 

**Table 1:** Characteristics and molecular epidemiology of VRSA, VISA, and hVISA isolates in South Asian countries.

Country	Study Period	Isolation Site	No. of Positive VRSA/Total	No. of Positive VISA/Total	No. of Positive hVISA/Total	Genetic Marker (*van*A/*van*B*/ica*A)	Molecular Typing	Reference
Pakistan	2020	Pus, skin wound, CSF	113/200	---	---	---	---	[69]
Pakistan	February 2017–March 2018	Pus swabs from diabetic foot ulcers, wounds, breast abscesses	565/6780	792/6780	---	---	---	[67]
Pakistan	January 2010–December 2010	Pus, urine, blood, vaginal swab, and other secretions	1/174	4/174	---	---	---	[66]
Pakistan	2016	Pus	22/110	5/110	---	---	---	[70]
Pakistan	2017–2018	Blood, body fluid, pus, skin wound	14/100	---	---	*van*A	---	[101]
Pakistan	2016	Pus from ear, skin wound	11/150	---	---	*van*A	---	[64]
Pakistan	2011	Blood	1/1	---	---	*van*A*, ica*A	---	[65]
Pakistan	2015	Wound, pus swab	5/51	---	---	---	---	[102]
Nepal		Blood, urine, sputum, catheter swab, pus, and body fluids	2/57	31/57	---	---	---	[103]
Nepal	2010	Lacrimal swabs from chronic dacryocystitis	22/27	---	---	---	---	[92]
Nepal	November 2011–May 2012	Urine, blood, and body fluids	---	4/45	---	---	---	[93]
India	January 1997–March 2000	Clinical isolates	---	---	5/80	---	---	[76]
India	August 2002–July 2005	Pus, urine, wound swabs, catheters, blood, sputum, and CSF	2/783	6/783	---	0	---	[104]
India	2016	Dental caries	27/150	---	---	13 *(van*A) 2(*van*B*)*	---	[105]
India	July 2010–September 2012	Pus, urine, wound swabs, catheters, blood, and sputum	7/115	53/115	---	---	---	[74]
India	March 2008–October 2008	Blood, urine and throat swabs, wounds, and ear swabs	7/358	16/358	---	6 *(van*A*)*	---	[106]
India	May 2013–October 2013	Clinical samples	3/100	12/100	6/100	---	---	[107]
India	July 2009–December 2012	Surgical site infection	0/267	3/267	---	---	---	[108]
India	January 2014–December 2016	Clinical samples	---	---	66/500	---	SCC*mec* III (8%), SCC*mec* IV (17.7%), SCC*mec* V (50%)	
India	July 2015–June 2016	Pus, respiratory tract, urine, blood, body fluids, and catheter tips	---	18/266	15/266	---	NA	[75]
India	February 2019–March 2020	Pus, tissue, blood, catheter	---	---	14/220	---	---	[80]
Sri Lanka	April 2002	Surgical site infection	---	1/1	---	---	---	[87]
Bangladesh	August 2010–July 2011	Clinical samples		114/122	---	---	---	
Bangladesh	January 2010–December 2011	Clinical samples	3/38	---	---	---	---	[109]
Bangladesh	July 2011–June 2012	Wound swabs	2/15	---	---	*van*B	---	[81]
Bangladesh	April 2012–January 2013	Burn wounds	---	16/29	---	---	---	[19]

## 4. Conclusions

Infections due to VISA/VRSA are associated with high morbidity and mortality, and an increase in the MIC creep of vancomycin has been found among clinical isolates during recent decades in different countries of South Asia. The subsequent increase in the vancomycin MIC trend is quite alarming for clinicians in the treatment of infections. The epidemiological investigation should be implemented by clinical laboratories for the determination of vancomycin non-susceptibility with accurate precision. Furthermore, long-term studies are needed to explore the resistance mechanism and epidemiological patterns of VISA/VRSA. Vancomycin in a clinical setting should be cautiously used to curtail the emergence and spread of resistant strains of *S. aureus*.

## Data Availability

Not applicable.
